# GBP5-triggered AIM2 inflammasome drives host defense and exacerbates disease severity during *Neospora caninum* infection

**DOI:** 10.1186/s13567-026-01769-z

**Published:** 2026-06-20

**Authors:** Mengge Chen, Xuancheng Zhang, Xiaocen Wang, Zhenzhen Liu, Boya Du, Xiaodan Yuan, Zhichao Sun, Sining Chen, Yanhui Yu, Pengtao Gong, Nan Zhang, He Li, Jianhua Li, Xu Zhang, Xin Li

**Affiliations:** 1 State Key Laboratory for Diagnosis and Treatment of Severe Zoonotic Infectious Diseases, Key Laboratory for Zoonosis Research of the Ministry of Education, Institute of Zoonosis, and College of Veterinary Medicine, Jilin University, Changchun, China; 2 Second Affiliated Hospital, Jilin University, Changchun, China; 3 Liaoning Center for Animal Disease Control and Prevention, Shenyang, China

**Keywords:** *Neospora caninum*, AIM2 inflammasome, GBP5, inflammatory response, parasite proliferation, tissue damage

## Abstract

*Neospora caninum* is a major cause of abortion in cattle worldwide, leading to substantial economic losses in the livestock industry. As no effective drug or vaccine is currently available, a deeper understanding of the host immune response against *N. caninum* is essential for developing effective control strategies. The absent in melanoma 2 (AIM2) inflammasome is involved in host defense and regulation of disease pathology, while its role in *N. caninum* infection remains unclear. This study shows that *N. caninum* activates the AIM2 inflammasome in wild-type (WT) murine peritoneal macrophage (PMϕs), characterized by increased expression of AIM2, pro-IL-1β, caspase-1 p20, and IL-1β p17, along with elevated IL-1β secretion and cell death rates. Guanylate-binding proteins (GBPs) are involved in regulating AIM2 inflammasome activation. Here, we observed that both GBP2 and GBP5 were upregulated by *N. caninum*, but only GBP5 overexpression played a functional role, as its overexpression enhanced, whereas its knockdown significantly attenuated AIM2 inflammasome in WT PMϕs, suggesting *N. caninum* activates the AIM2 inflammasome in a GBP5-dependent manner. To further investigate the role of AIM2 in parasite infection, *AIM2*^*−*/*−*^ mice were used. In *AIM2*^*−*/*−*^ PMϕs, inflammasome activation was reduced, accompanied by increased parasite proliferation. In vivo, *N. caninum*-infected *AIM2*^*−*/*−*^ mice exhibited higher parasite loads but showed increased survival rates, reduced macrophage recruitment, decreased levels of IFN-γ, and IL-18, and alleviated pathological damage. In summary, our findings demonstrate that the AIM2 inflammasome is activated by *N. caninum* in a GBP5-dependent manner and plays a dual role by restricting parasite proliferation while exacerbating disease pathology.

**Supplementary Information:**

The online version contains supplementary material available at 10.1186/s13567-026-01769-z.

## Introduction

*Neospora caninum* is an intracellular protozoan that causes neosporosis, a globally significant livestock disease [1]. It is a major cause of abortion and stillbirth in ruminants such as cattle and sheep, imposing a substantial economic burden on the beef and dairy industries worldwide [1–3]. Despite its economic and veterinary importance, no effective drugs or commercial vaccines are currently available to control neosporosis, making it an urgent and unresolved challenge in veterinary medicine. Host innate immunity serves as the first line of defense against pathogens through the recognition of pathogen-associated molecular patterns (PAMPs) and damage-associated molecular patterns (DAMPs) by pattern recognition receptors (PRRs) [4, 5]. Therefore, a deeper understanding of host–parasite interactions is crucial for developing novel immunomodulatory strategies against neosporosis.

AIM2, a cytosolic DNA sensor that recognizes double-stranded DNA, is a central component of innate immunity. Upon activation, AIM2 assembles into a multiprotein complex with ASC and pro-caspase-1 via PYD–PYD and CARD–CARD interactions, driving caspase-1 cleavage, pyroptosis, and the secretion of the proinflammatory cytokines IL-1β and IL-18 [6]. This pathway plays a pivotal role in host defense against a broad range of pathogens, including *Streptococcus pneumoniae*, *Listeria monocytogenes*, *Brucella abortus*, murine cytomegalovirus, and *Toxoplasma gondii* [7–11]. While the protective functions of the AIM2 inflammasome are well characterized, a limited number of studies have revealed its pathological role. AIM2 activation exacerbates cutaneous pathology in *Leishmania (V.) braziliensis* infection [12], and mediates inflammatory lung injury during influenza A virus infection [13]. However, the role of AIM2 inflammasome in *N. caninum* infection remains to be elucidated.

Guanylate-binding proteins (GBPs) are an interferon (IFN)-inducible subfamily of dynamin-related large GTPases and are highly conserved among mammals [14]. To date, the most extensively characterized GBPs are those of humans and mice; the human genome encodes 7 GBPs, the mouse genome encodes 11 functional murine GBPs (mGBPs) and 2 pseudogenes [15]. A well-established function of GBPs is their ability to directly recognize and perforate microbial membranes through nucleotide-dependent conformational changes and specific targeting motifs [16, 17]. For instance, mGBP1, mGBP2, mGBP3, and mGBP5 localize to the membranes of diverse intracellular pathogens, including influenza A virus, *Shigella flexneri*, and *Chlamydia trachomatis*, where they exert antimicrobial effects through membrane disruption [18–20]. Similarly, multiple GBPs disrupt the *T. gondii* parasitophorous vacuole membrane (PVM) and plasma membrane to inhibit replication [21, 22]. Beyond direct membranolysis, GBPs regulate inflammasome activation [23]. mGBP2 and mGBP5 promote AIM2 inflammasome activation by releasing *F. novicida* DNA into the cytosol [24], and human GBP5 drives NLRP3 inflammasome assembly and IL-1β/IL-18 secretion [25].

GBPs have also been identified in livestock species, including cattle and sheep, and they share high structural and sequence homology with human and murine GBPs [26, 27]. The bovine genome encodes GBP1, GBP2, GBP4, GBP5, and GBP6 [26, 28–30], and bovine GBPs are responsive to pathogen challenge. GBP5 is upregulated in bovine herpesvirus 1-infected cells and enhances host resistance [26], while GBP4 and GBP6 are induced by *Mycobacterium bovis* and *M. avium* subsp. *paratuberculosis*, respectively [28, 29]. In contrast, information on GBPs in sheep and dogs remains limited. The sheep genome harbors GBP6 [27], and the dog genome encodes GBP1, GBP5, and GBP6 (NCBI Gene IDs: 490172, 490170, and 490169, respectively), with current knowledge derived primarily from genomic annotation and structural prediction, but functional validation is lacking. Notably, our prior work identified mGBP2 and mGBP5 as the most markedly induced mGBPs in *N. caninum*-infected mouse PMϕs [31]. Whether GBPs mediate AIM2 inflammasome activation during *N. caninum* infection remains unknown.

Here, we report that GBP5-mediated activation of the AIM2 inflammasome plays a pivotal role during *N. caninum* infection. Mechanistically, GBP5 specifically localizes to the *N. caninum* membrane, inducing membranolysis, which triggers AIM2 inflammasome activation and the release of IL-1β and IL-18. Notably, *AIM2* deficiency markedly attenuates inflammasome activation, enhances parasite proliferation, and alleviates tissue pathology in mice. These findings reveal a dual role for the GBP5–AIM2 axis in balancing host defense and immunopathology during neosporosis, offering new insights into the innate immune regulation of veterinary parasitic infections and potential therapeutic targets for disease intervention.

## Materials and methods

### Animals

Wild-type (WT) C57BL/6 mice (5–8 weeks old) were purchased from the Experimental Animal Center (Changsheng, Benxi, China), and *AIM2*-deficient (*AIM2*^*−*/*−*^) mice on the C57BL/6 genetic background were obtained from the Jackson Laboratory (Bar Harbor, ME, USA). All mice were housed in a sterile, independent ventilation system under specific pathogen-free conditions.

### Cell and parasite culture

Vero cells were cultured in Roswell Park Memorial Institute (RPMI)−1640 medium (VivaCell, Shanghai, China) with 5% fetal bovine serum (FBS; VivaCell, Shanghai, China) and 1% penicillin–streptomycin (VivaCell, Shanghai, China) at 37 °C under 5% CO_2_. *N. caninum* tachyzoites (Nc-1 strain, ATCC: 50977) were cultured in Vero cells using RPMI-1640 supplemented with 1% FBS at 37 °C, and were harvested when approximately 80% of Vero cells were lysed. Vero cell debris was removed by gradient density centrifugation with 10 mL 40% Percoll (GE Healthcare, Uppsala, Sweden) solution, centrifuged at 2000 *g* for 30 min, then *N. caninum* tachyzoites were harvested and washed twice by centrifugation at 900 *g* for 5 min with RPMI-1640 [32], and the number of *N. caninum* tachyzoites was counted using a hemocytometer under a microscope.

### Mouse peritoneal macrophage (PMϕs) culture

Female WT and *AIM2*^*−*/*−*^ mice were injected intraperitoneally with 2 mL sterile 2.98% Difo Fluid Thioglycolate medium (BD, Franklin Lakes, USA) and euthanized at 3 days post-injection, then the peritoneal cavity was lavaged with 6 mL ice-cold phosphate-buffered saline (PBS). The collected lavage fluid was centrifuged at 1000 *g* for 10 min to collect PMϕs. PMϕs were counted and seeded into 6-well plates at a density of 3 × 10^6^ cells/well, and cultured with RPMI-1640 supplemented with 10% FBS and 1% penicillin–streptomycin at 37 °C under 5% CO_2_.

### *N. caninum* infection experiments in vitro

For the detection of AIM2 expression level, PMϕs were infected with *N. caninum* tachyzoites at a multiplicity of infection (MOI) = 1 (parasite:cell) for different time points (6, 12, 18, and 24 h), or at different MOIs (1, 3, and 5) for 24 h. Cells treated with medium alone served as the negative control (the medium group). For the positive control (lipopolysaccharide [LPS] + poly(dA:dT)), PMϕs were pretreated with 100 ng/mL LPS (Sigma, Shanghai, China) for 2 h, washed twice with PBS, and then stimulated using AIM2 inflammasome inducer poly(dA:dT) (1 μg/mL, Invivogen, San Diego, USA) for 24 h. Cells were collected, and AIM2 messenger RNA (mRNA) and protein levels were detected by quantitative real-time polymerase chain reaction (qPCR) and western blot.

For the detection of AIM2 inflammasome activation, WT and *AIM2*^*−*/*−*^ PMϕs were infected with *N. caninum* (MOI = 1) for 24 h, then cell culture supernatants were collected for detection of caspase-1 p20 and IL-1β p17 by western blot, IL-1β secretion by enzyme-linked immunosorbent assay (ELISA), and cell death by lactate dehydrogenase (LDH) release assay. Cell lysates were collected for analysis of pro-caspase-1 and pro-IL-1β expression by western blot assay, and the determination of the number of *N. caninum* tachyzoites by qPCR.

For the detection of GBP2 and GBP5 expression levels, PMϕs were infected with *N. caninum* at various MOIs (1, 3, or 5) for 24 h. GBP2 and GBP5 protein levels were detected by western blot.

For the detection of the roles of GBP5 in *N. caninum*-induced AIM2 inflammasome activation, PMϕs were transfected with pcDNA3.1 empty vector (1 μg), pcDNA3.1-GBP5 plasmids (1 μg; constructed in our laboratory), control small interfering RNA (siRNA) (sicontrol) (1 μg), or GBP5-siRNA (siGBP5, 1 μg; RiboBio, Guangzhou, China) using Lipofectamine 2000 (Invitrogen, CA, USA) according to the manufacturer’s instructions. After 24 h of transfection, cells were infected with *N. caninum* (MOI = 1) for 24 h. Cell culture supernatants were collected for detection of caspase-1 p20 and IL-1β p17 by western blot, IL-1β secretion by ELISA, and cell death by LDH release assay. Cell lysates were collected for analysis of GBP5, AIM2, pro-caspase-1, and pro-IL-1β expression by western blot. The membrane structure of the *N. caninum* tachyzoites was observed by transmission electron microscopy (TEM).

### *N. caninum* infection in vivo

For survival analysis, WT and *AIM2*^*−*/*−*^ mice (*n* = 10 per group) were intraperitoneally injected with 2 × 10^7^ *N. caninum* tachyzoites. Mortality was monitored daily for 30 days post-infection, and survival curves were generated.

WT and *AIM2*^*−*/*−*^ mice were randomly divided into two experimental groups (*n *= 5 per group). Mice were intraperitoneally injected with 100 μL sterile PBS as the negative control group (PBS group). Mice were intraperitoneally injected with 2 × 10^7^ tachyzoites suspended in 100 μL PBS as the *N. caninum*-infected group (*N. c* group). All mice were humanely euthanized at 5 days post-infection (dpi) in accordance with institutional animal welfare guidelines, and tissues (heart, liver, spleen, lung, kidney, and brain) were collected for immunohistochemical analysis of AIM2 and F4/80 expression, parasite burden quantification, and histopathological examination. Blood was collected via cardiac puncture, and serum was isolated for cytokine measurement by ELISA [33].

### Determination of AIM2 mRNA by qPCR

Total RNA was extracted from PMϕs using TRIzol reagent (Epizyme, Shanghai, China). RNA concentration and purity were assessed using a Nanodrop ND-2000 spectrophotometer (Thermo, MA, USA), and complementary DNA (cDNA) was synthesized from 1 μg of total RNA using the PrimeScript RT reagent Kit (Takara, Dalian, China). Subsequently, qPCR was performed on a LightCycler 480 II system (Roche, Mannheim, Germany) using 2 × Universal SYBR Green qPCR Master Mix (Servicebio, Wuhan, China) to measure the mRNA expression levels of AIM2. The thermal cycling conditions were as follows: initial denaturation at 95 °C for 30 s, followed by 40 cycles of 95 °C for 15 s and 60 °C for 30 s. A melting curve analysis was conducted at the end of each run to verify amplification specificity. All samples were run in triplicate, and the mean cycle threshold (Ct) values were used for analysis. To validate the stability of candidate housekeeping genes during *N. caninum*-infected PMϕs, the expression stability of five genes (glyceraldehyde 3-phosphate dehydrogenase [GAPDH], TATA-box binding protein [Tbp], peptidylprolyl isomerase A [Ppia], hypoxanthine–guanine phosphoribosyltransferase [Hprt], and ribosomal protein L13a [RPL13a]) was evaluated using BestKeeper (version 1), and GAPDH was confirmed to be stably expressed across all experimental conditions and was therefore selected as the reference gene for this study [34]. The relative expression of AIM2 was normalized to GAPDH, and fold changes were calculated using the 2^−ΔΔCt^ method. Primer sequences used are listed in Additional file 1.

### Western blot analysis

For the detection of secreted proteins, cell culture supernatants were precipitated with methanol and chloroform, and then the precipitated proteins were dissolved in 1% sodium dodecyl sulfate (SDS) buffer to obtain protein samples. For cellular proteins, PMϕs were lysed with 100 µL RIPA lysis buffer (Boster, Wuhan, China) containing protease inhibitor cocktail. Protein concentrations were determined using a bicinchoninic acid (BCA) protein assay kit (Beyotime, Shanghai, China).

Protein samples (30 μg) were separated by SDS–polyacrylamide gel electrophoresis (PAGE) and transferred to polyvinylidene difluoride (PVDF) membrane (Millipore, Bedford, USA). Membranes were blocked with protein-free rapid blocking solution (EpiZyme, Shanghai, China) for 30 min at room temperature (RT), then incubated with rabbit anti-AIM2 (1:2000, Abcam, Cambridge, UK), goat anti-IL-1β (1:2000, R&D, Minneapolis, MN, USA), mouse anti-caspase-1 (p20) (1:1000, Adipogen, Liestal, Switzerland), or mouse anti-GAPDH (1:20 000, Proteintech, Wuhan, China) antibodies at 4 ℃ overnight [11]. After membranes were washed with Tris-buffered saline with Tween-20 (TBST) three times, they were incubated with horseradish peroxidase (HRP) anti-rabbit IgG antibodies, HRP anti-goat IgG antibodies, or HRP anti-mouse IgG antibodies (1:5000, Proteintech, Wuhan, China) at RT for 1 h. Then, membranes were washed with TBST three times, and protein bands were visualized using an enhanced chemiluminescence (ECL) substrate (EpiZyme, Shanghai, China) on a Clinx ChemiScope Series imaging system (Clinx, Shanghai, China). Protein quantification analysis was performed using ImageJ.

### Immunofluorescence assay (IFA)

For detection of AIM2 inflammasome activation and the subcellular localization of GBP2 and GBP5, WT PMϕs were seeded on sterile coverslips in 24-well plates and infected with *N. caninum* (MOI = 1) for 24 h. Cells were then fixed with 4% paraformaldehyde for 30 min at RT, washed three times with PBS, and permeabilized with 0.5% Triton X-100 for 20 min at RT. After washing three times, cells were blocked with 3% bovine serum albumin (BSA) for 30 min.

For AIM2 and ASC colocalization, cells were incubated overnight at 4 °C with rabbit anti-AIM2 antibody (1:200, eBioscience, San Diego, USA) and mouse anti-ASC antibody (1:200, Santa Cruz, CA, USA). For GBP localization, cells were incubated overnight at 4 °C with rabbit anti-GBP2 or anti-GBP5 antibody (1:200, Proteintech, Wuhan, China) and mouse anti-NcSAG1 antibody (1:200, homemade polyclonal antibody). Following primary antibody incubation, cells were washed three times with PBS and incubated with the CoraLite488-conjugated recombinant rabbit anti-mouse antibody and CoraLite594-conjugated recombinant goat anti-rabbit antibody (1:200, Proteintech, Wuhan, China) for 1 h at RT in the dark. After washing, slides were sealed with DAPI containing an anti-fluorescence quench agent (Beyotime, Shanghai, China). Finally, images were acquired by FV3000 (Olympus, Tokyo, Japan), five fields were analyzed in each sample, and three independent experiments were performed.

### Lactate dehydrogenase (LDH) release assay

Cell culture supernatants were collected, and LDH release was measured using a commercial LDH cytotoxicity assay kit (Beyotime, Shanghai, China) in accordance with the manufacturer’s instructions. Absorbance was read at 490 nm using a microplate reader.

### Enzyme-linked immunosorbent assays (ELISA)

The levels of IL-1β, IL-12 p40, IL-18, TNF-α, and IFN-γ in cell culture supernatants and mouse serum were quantified using commercial ELISA kits (Invitrogen, CA, USA) according to the manufacturer’s protocols. Absorbance was read at 450 nm using a microplate reader.

### The infection rate and proliferation of *N. caninum* assay

WT and *AIM2*^*−*/*−*^ PMϕs (5 × 10^5^ cells/well) were seeded on coverslips in 24-well plates and infected with *N. caninum* tachyzoites (MOI = 1). After 2 h of infection, cells were washed twice with sterile PBS and cultured for an additional 24 h [32]. The infection rate and parasite proliferation were detected by IFA and Giemsa staining.

For the IFA assay, PMϕs were fixed with 4% paraformaldehyde for 30 min, washed three times with sterile PBS, and permeabilized with 0.5% Triton X-100 at RT for 20 min. After washing three times, the cells were incubated with 3% BSA for 30 min, then incubated with rabbit anti-NcSAG1 polyclonal antibody (1:200) at 4 °C overnight. After washing three times, cells were incubated with CoraLite594-conjugated recombinant goat anti-rabbit antibody (1:200, Proteintech, Wuhan, China) for 1 h at RT in the dark. After washing, slides were sealed with DAPI containing anti-fluorescence quench agent. Finally, images were acquired using a FV3000 confocal microscope.

For Giemsa staining, cells grown on coverslips were fixed with 4% paraformaldehyde for 10 min at RT. Following fixation, cells were stained using a commercial Giemsa staining kit according to the manufacturer’s protocol. The stained coverslips were then rinsed twice under running water, mounted on glass slides, and examined under a light microscope (BX43F, Olympus, Tokyo, Japan) for observation and image acquisition.

For the infection rate, five randomly selected non-overlapping fields were captured in each coverslip, and the percentage of infected cells was calculated by counting the total number of cells and the number of infected cells in each image of IFA and Giemsa staining. For parasite proliferation, three randomly selected fields were captured in each coverslip, and the number of tachyzoites per parasitophorous vacuole was quantified. At least 100 vacuoles per sample were counted to evaluate parasite proliferation in each image of IFA and Giemsa staining [35].

### Quantification of *N. caninum* burden by qPCR

To quantify the number of *N. caninum* tachyzoites in PMϕs and tissue samples, DNA was extracted from infected PMϕs and tissue samples using a commercial tissue-cell genomic DNA extraction kit (Tiangen, Beijing, China). A standard curve was generated using genomic DNA extracted from a known number (1 × 10^8^) of purified *N. caninum* tachyzoites, which was serially diluted tenfold to create eight standards. For each sample, 200 ng (PMϕs) or 500 ng (tissues) of total DNA was used as template for qPCR. qPCR was performed using SYBR Green Master Mix (Servicebio, Wuhan, China) on a LightCycler 480 II system (Roche, Mannheim, Germany) with the following primers targeting the Nc5 gene of *N. caninum*: Nc5-forward 5′-ACTGGAGGCACGCTGAACAC-3′, Nc5-reverse 5′-AACAATGCTTCGCAAGAGGAA-3′. Thermal cycling conditions were: initial denaturation at 95 °C for 30 s, followed by 40 cycles of 95 °C for 15 s and 60 °C for 30 s. Parasite numbers in experimental samples were calculated by interpolating from the standard curve [36].

### Transmission electron microscopy (TEM)

WT PMϕs were transfected with pcDNA3.1-GBP5 plasmid (1 μg) or siGBP5 (1 μg) using Lipofectamine 2000. After 24 h of transfection, cells were infected with *N. caninum* (MOI = 1) for an additional 24 h. Following infection, the culture medium was removed and replaced with electron microscopy fixative. Cells were fixed in the dark at RT for 5 min, then gently detached using a cell scraper in a unidirectional manner. The cell suspension was transferred to a centrifuge tube and centrifuged at 1000 *g* for 5 min. The fixative was discarded and replaced with fresh fixative, followed by an additional 30 min of fixation at room temperature in the dark. The fixed cell pellets were then processed for resin embedding. Ultrathin sections (60–80 nm) were prepared, stained with uranium and lead salts, and imaged using a TEM (HITACHI HT7700). For each sample, five fields of view were analyzed, and three independent experiments were performed.

### Immunohistochemical (IHC) staining

Tissue samples were collected, fixed in 4% paraformaldehyde, embedded in paraffin, and sectioned into 5-μm-thick slices. Tissue sections were placed in citrate buffer and boiled for 30 min after routine dewaxing, cooled to RT and washed with PBS, then incubated with rabbit-anti-AIM2 antibody (1:200, eBioscience, San Diego, USA), rabbit-anti-F4/80 antibody (1:200, Affinity, Cincinnati, USA), and DAB staining (Solarbio, Beijing, China) according to the manufacturer’s instructions for the UltraSensitive SP (mouse/rabbit) IHC Kit (Maixin, Fuzhou, China). Images were captured by two independent laboratory technicians blinded to the experimental groups using a light microscope, and protein expression was quantified using ImageJ software.

### Histopathological observation

Tissue samples were collected, fixed in 4% paraformaldehyde, embedded in paraffin, and sectioned into 5-μm-thick slices. Tissue sections were deparaffinized in xylene, rehydrated through a graded ethanol series, and stained with hematoxylin and eosin (H&E). Pathological changes were observed by two independent pathologists blinded to the experimental groups, and images were captured using a light microscope.

### Statistical analysis

All experiments were performed with at least three independent biological replicates, and each sample was assessed in technical triplicates. Data are presented as mean ± standard deviation (SD) from three independent experiments. Statistical analysis was performed with GraphPad Prism 6.01 (GraphPad Software, CA, USA). Comparisons between two groups were evaluated using Student’s *t*-test, while multiple-group comparisons were followed by one-way analysis of variance (ANOVA). Survival curves were generated using the Kaplan–Meier method, and statistical significance between groups was assessed by the log-rank test. Significance levels are denoted as follows: no significant difference (ns) *P* > 0.05, *P < 0.05, **P < 0.01, ***P < 0.001, and ****P < 0.0001.

## Results

### *N. caninum* infection upregulates AIM2 expression in mouse PMϕs

**Figure 1:**
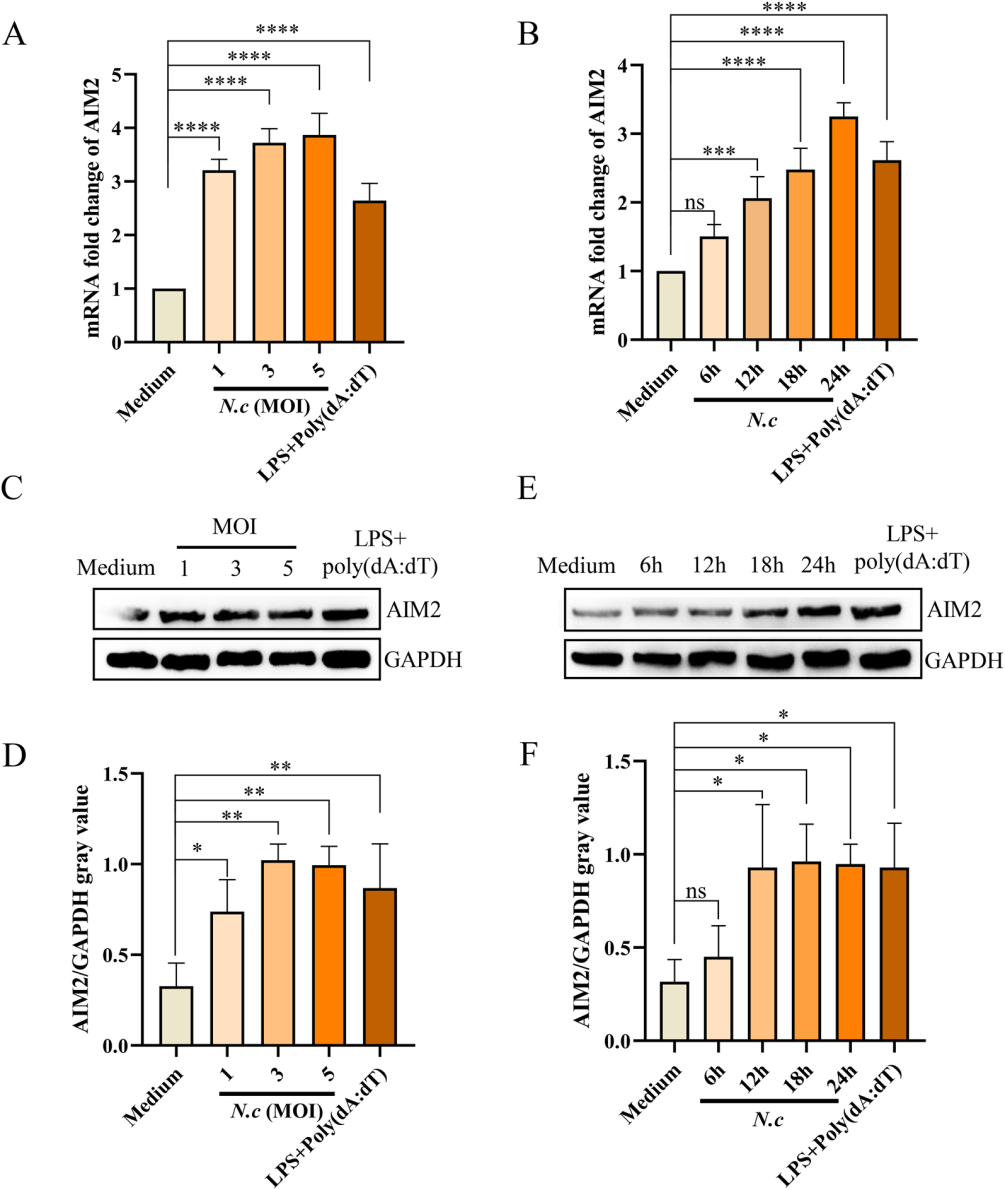
**AIM2 expression is upregulated in *N. caninum*-infected mouse macrophages.** Mouse PMϕs were infected with *N. caninum* at the indicated multiplicities of infection (MOI = 1, 3, or 5) for 24 h, or for the indicated time points (6, 12, 18, or 24 h) at MOI = 1. Cells were treated with medium alone, which served as the negative control (the medium group), and cells were treated with LPS (100 ng/mL for 2 h) + poly(dA:dT) (1 μg/mL) for 24 h, which served as the positive control. **A, B** AIM2 mRNA level was measured by qPCR. **C, E** AIM2 protein expression was measured by western blot. **D, F** AIM2 protein level was quantified using ImageJ. Data are displayed as mean ± SD of three independent experiments (*n *= 3). No significant difference (ns), *P < 0.05, **P < 0.01, ***P < 0.001, and **** *P* < 0.0001 (one-way ANOVA).

To determine whether AIM2 expression is altered during *N. caninum* infection, mouse PMϕs were infected with *N. caninum* at various MOIs and for different times. AIM2 transcription and protein expression were then detected by qPCR and western blot. qPCR analysis revealed that *AIM2* mRNA levels were significantly increased in the *N. caninum*-infected group compared with the medium group, and this upregulation occurred in a dose- and time-dependent manner (Figure 1A, B). Consistently, western blot analysis demonstrated that the expression level of AIM2 protein was also increased following *N. caninum* infection, with the increase correlating with both infection dose and duration (Figure 1C–F). These results suggested that *N. caninum* infection promoted AIM2 expression in mouse PMϕs.

## *N. caninum* activates the AIM2 inflammasome in mouse PMϕs

**Figure 2:**
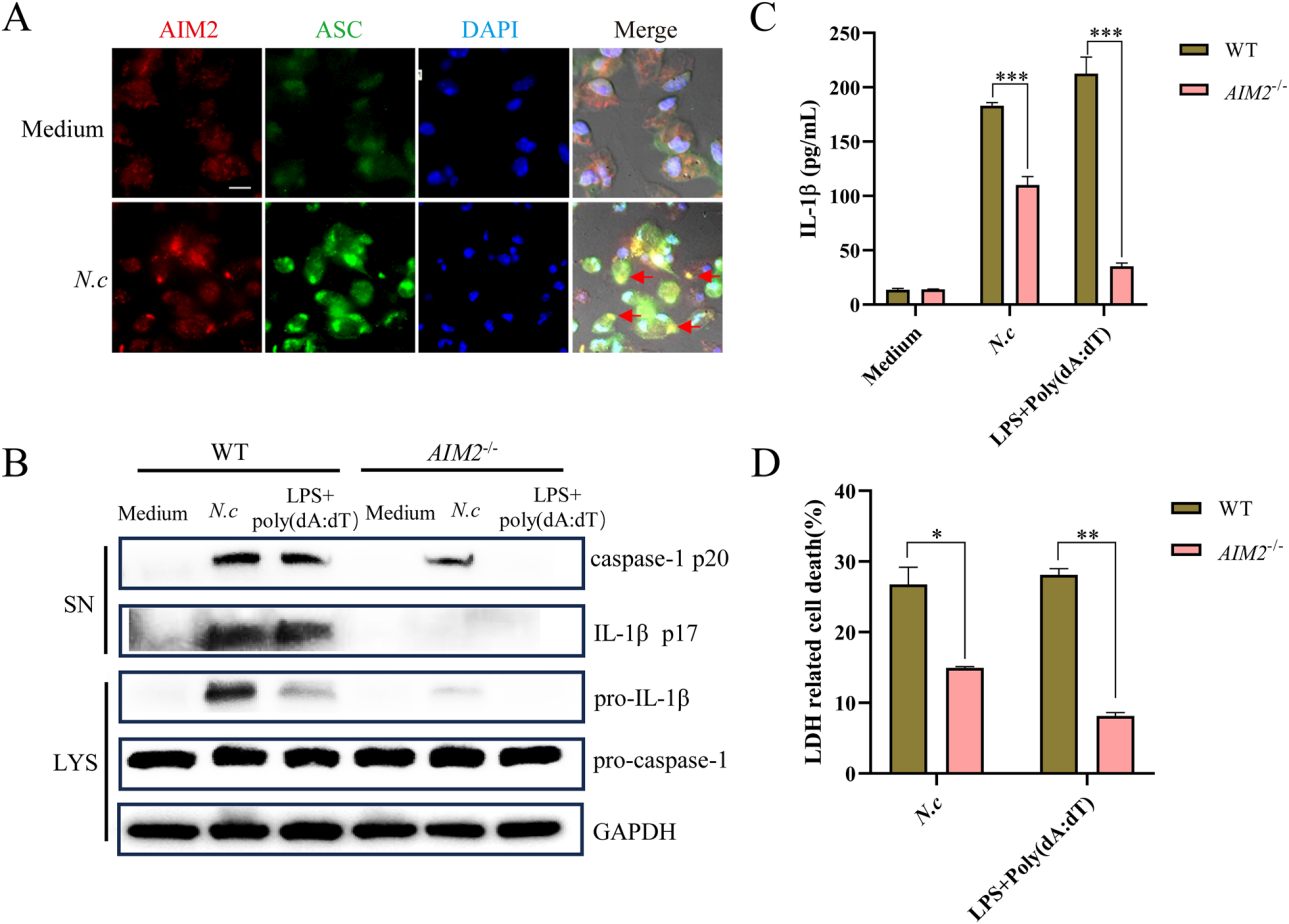
***Neospora caninum*** **infection induces AIM2 inflammasome activation in mouse macrophages**. WT and *AIM*2.^*−*/*−*^ PMϕs were infected with *N. caninum* for 24 h. **A** The colocalization of AIM2 and ASC (indicated by red arrows) was detected by IFA, scale bars = 20 μm. **B** The levels of active caspase-1 (p20) and mature IL-1β (p17) in supernatants (SN), as well as the expression of pro-IL-1β and pro-caspase-1 in cell lysates (LYS) were detected by western blot. **C** The level of IL-1β secretion in the supernatant was detected by ELISA. **D** Cell death rate was evaluated by LDH assay. Data are displayed as mean ± SD of three independent experiments (*n *= 3). *P < 0.05, **P < 0.01, ***P < 0.001 (*t*-test).

To investigate whether the AIM2 inflammasome is activated in *N. caninum*-infected PMϕs, we examined the assembly of the AIM2 inflammasome complex by IFA and assessed the expression of key inflammasome effector proteins by western blot. IFA analysis revealed that AIM2 and ASC were diffusely distributed in the cytoplasm in the medium control group, whereas both proteins formed aggregates with strong colocalization in *N. caninum*-infected cells (Figure 2A), a pattern similar to that observed in cells treated with the known AIM2 inflammasome inducer LPS + poly(dA:dT) in a previous study [37], indicating inflammasome complex formation. Furthermore, *N. caninum* infection significantly increased the protein levels of IL-1β p17, caspase-1 p20, and pro-IL-1β (Figure 2B); enhanced IL-1β secretion (*P* < 0.001) (Figure 2C); and promoted LDH release (Figure 2D) in WT PMϕs, while*AIM2* deficiency markedly attenuated these *N. caninum*-induced responses (Figure 2B–D). Collectively, these findings demonstrated that *N. caninum* infection triggered the activation of the AIM2 inflammasome in mouse PMϕs.

To address potential redundancy between inflammasome pathways, we also evaluated whether *AIM2* deficiency leads to compensatory changes in NLRP3 inflammasome activation. IFA analysis revealed no significant difference in the NLRP3 speckle-forming cells between WT and *AIM2*^*−*/*−*^ PMϕs following *N. caninum* infection (Additional File 2 A). Consistently, western blot analysis showed that NLRP3 protein levels were comparable between the two genotypes (Additional File 2B, C). These results indicated that AIM2 deficiency does not lead to compensatory upregulation of the NLRP3 inflammasome at the protein level.

## GBP5 targets *N. caninum* membrane and promotes AIM2 inflammasome activation

**Figure 3:**
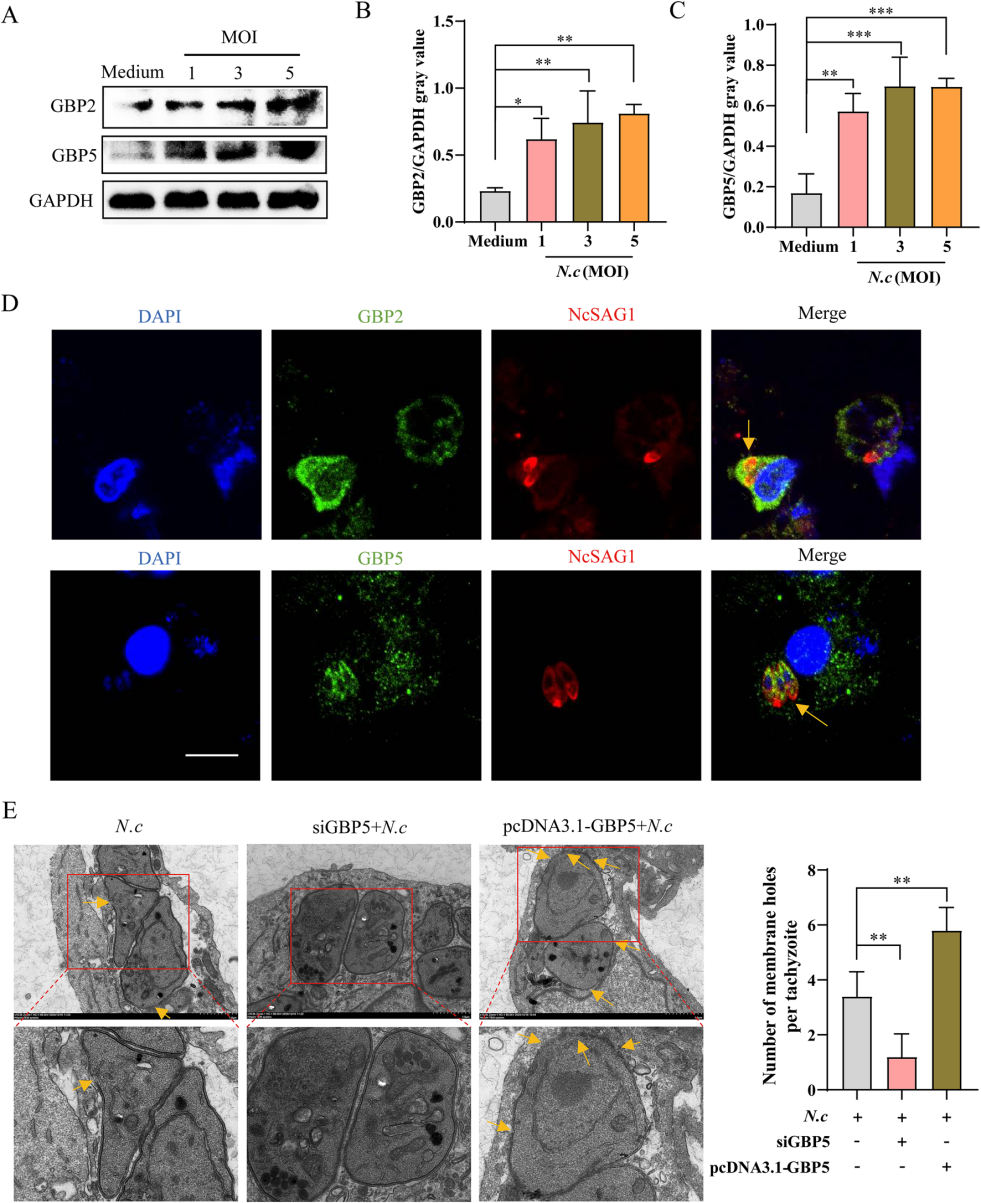
**GBP5 targets and disrupts the *N. caninum* membrane.** **A** PMϕs were infected with *N. caninum* at various MOIs (1, 3, or 5) for 24 h. GBP2 and GBP5 protein levels were detected by western blot. **B, C** GBP2 and GBP5 protein levels were quantified using ImageJ. **D** The subcellular localization of GBP2 and GBP5 was detected by IFA. GBP2 or GBP5 (green), NcSAG1 (*N. caninum* membrane, red), scale bars = 10 μm. **E** PMϕs were transfected with pcDNA3.1-GBP5 (1 μg) plasmids or siGBP5 for 24 h, followed by infection with *N. caninum* (MOI = 1) for 24 h. The membrane structure of *N. caninum* was observed by TEM at 8000× magnification; random fields were selected from each specimen, and membrane holes were counted. Orange arrows indicate membrane hole sites. Data are displayed as mean ± SD of three independent experiments (*n *= 3). *P < 0.05, **P < 0.01, ***P < 0.001 (one-way ANOVA).

Our previous study examined the mRNA levels of GBP1–11 in *N. caninum*-infected PMϕs, and GBP2 and GBP5 showed the most significant upregulation. Given that GBPs can target and disrupt the parasitophorous vacuole or membrane of intracellular pathogens to regulate AIM2 inflammasome activation [24, 38], we investigated whether GBP2 and GBP5 recognize the *N. caninum* membrane by examining their protein expression levels and subcellular localization in infected PMϕs. Western blot analysis showed that GBP2 and GBP5 protein levels were increased in *N. caninum*-infected groups compared with the medium group (MOI = 1, *P* < 0.05; MOI = 3, *P* < 0.01; MOI = 5, *P* < 0.01) (Figure 3A–C). IFA revealed that GBP2 was mainly located in the cytoplasm with minimal colocalization with NcSAG1 (a *N. caninum* membrane protein). Nevertheless, GBP5 exhibited robust colocalization with NcSAG1 (Figure 3D), suggesting that GBP5 is located on the parasite membrane. TEM further demonstrated that the parasitic membrane was disrupted in the *N. caninum*-infected group, and overexpression of GBP5 increased the number of membrane holes on the tachyzoite (*P* < 0.01), whereas knockdown of GBP5 reduced membrane hole formation (*P* < 0.01) (Figure 3E). Collectively, these results indicated that GBP5 specifically targeted and disrupted the *N. caninum* membrane.

**Figure 4:**
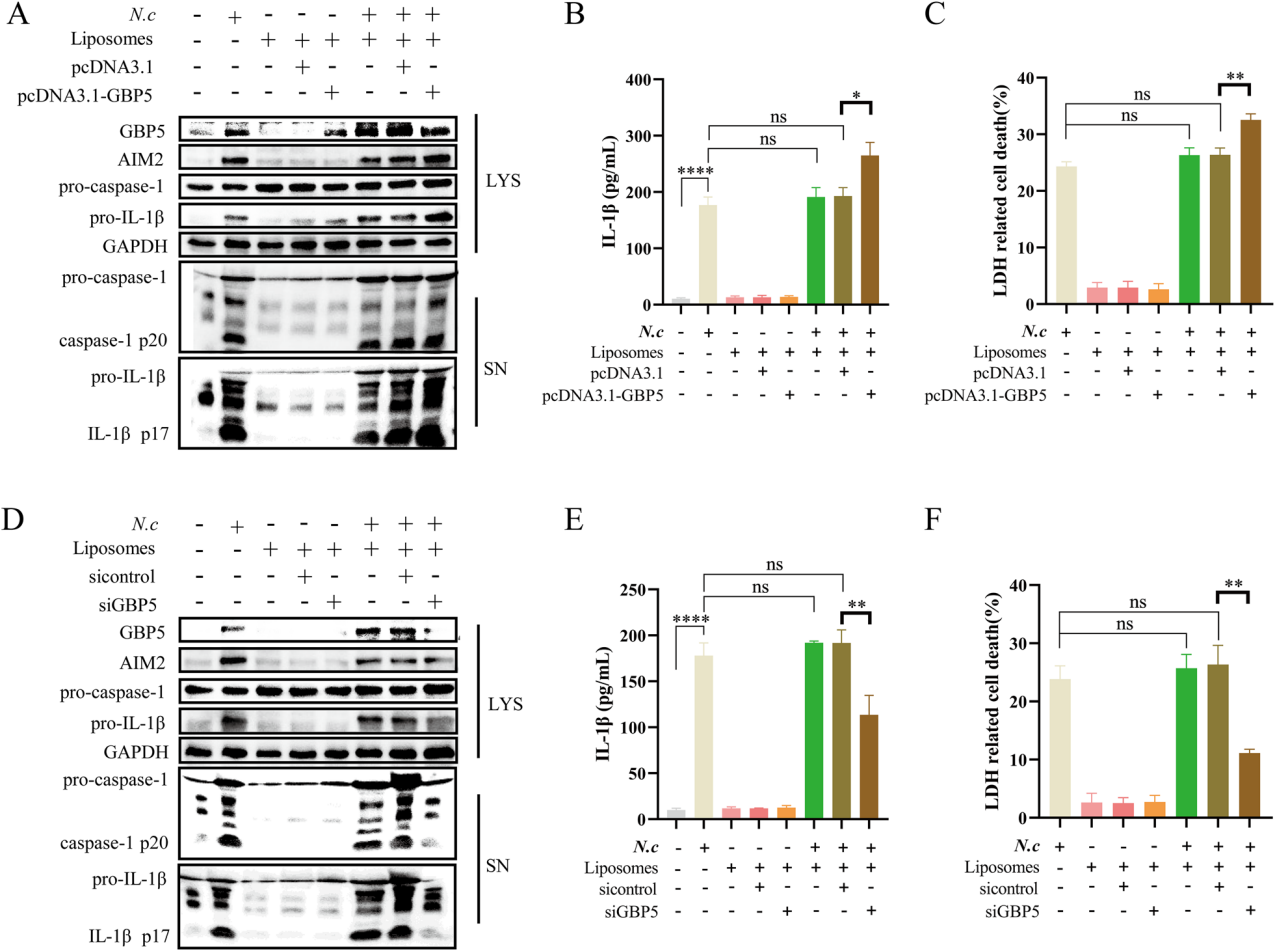
**GBP5 promotes *N. caninum* infection-induced AIM2 inflammasome activation in mice macrophages.** **A** PMϕs were transfected with pcDNA3.1 (1 μg, empty vector control) or pcDNA3.1-GBP5 (1 μg) for 24 h, then infected with *N. caninum* (MOI = 1) for 24 h. The protein levels of GBP5, AIM2, pro-IL-1β, pro-caspase-1, the caspase-1 p20, and IL-1β p17 were detected by western blot. **B** The level of IL-1β secretion in the supernatant was detected by ELISA. **C** Cell death rate was evaluated by LDH assay. **D** PMϕs were transfected with sicontrol (1 μg) or GBP5-siRNA (siGBP5, 1 μg) for 24 h, then infected with *N. caninum* (MOI = 1) for 24 h. The protein levels of GBP5, AIM2, pro-IL-1β, pro-caspase-1, the caspase-1 p20, and IL-1β p17 were detected by western blot. **E** The level of IL-1β secretion in the supernatant was detected by ELISA. **F** Cell death rate was evaluated by LDH assay. Data are displayed as mean ± SD of three independent experiments (*n *= 3). *P < 0.05, **P < 0.01, ***P < 0.001, and **** *P* < 0.0001 (*t*-test). SN, supernatant; LYS, cell lysate.

To further investigate the role of GBP5 in *N. caninum*-induced AIM2 inflammasome, PMϕs were subjected to GBP5 overexpression or knockdown prior to *N. caninum* infection. Compared with the empty vector control group (pcDNA3.1 + *N. caninum*), overexpression of GBP5 significantly upregulated *N. caninum*-induced protein levels of GBP5, AIM2, pro-IL-1β, IL-1β p17, and caspase-1 p20 (Figure 4A and Additional file 3A–D); enhanced IL-1β secretion (*P* < 0.05) (Figure 4B); and increased LDH release (*P* < 0.01) (Figure 4C). Conversely, knockdown of GBP5 markedly reduced the *N. caninum*-induced protein levels of these molecules (Figure 4D and Additional file 3E–H) and diminished IL-1β secretion (*P* < 0.01) (Figure 4E) and LDH release (*P* < 0.01) (Figure 4F) compared with the control siRNA group (sicontrol + *N. caninum*). These results indicated that GBP5 targeted the *N. caninum* membrane and promoted AIM2 inflammasome activation.

## AIM2 inflammasome restricts *N. caninum* proliferation in mouse PMϕs

**Figure 5:**
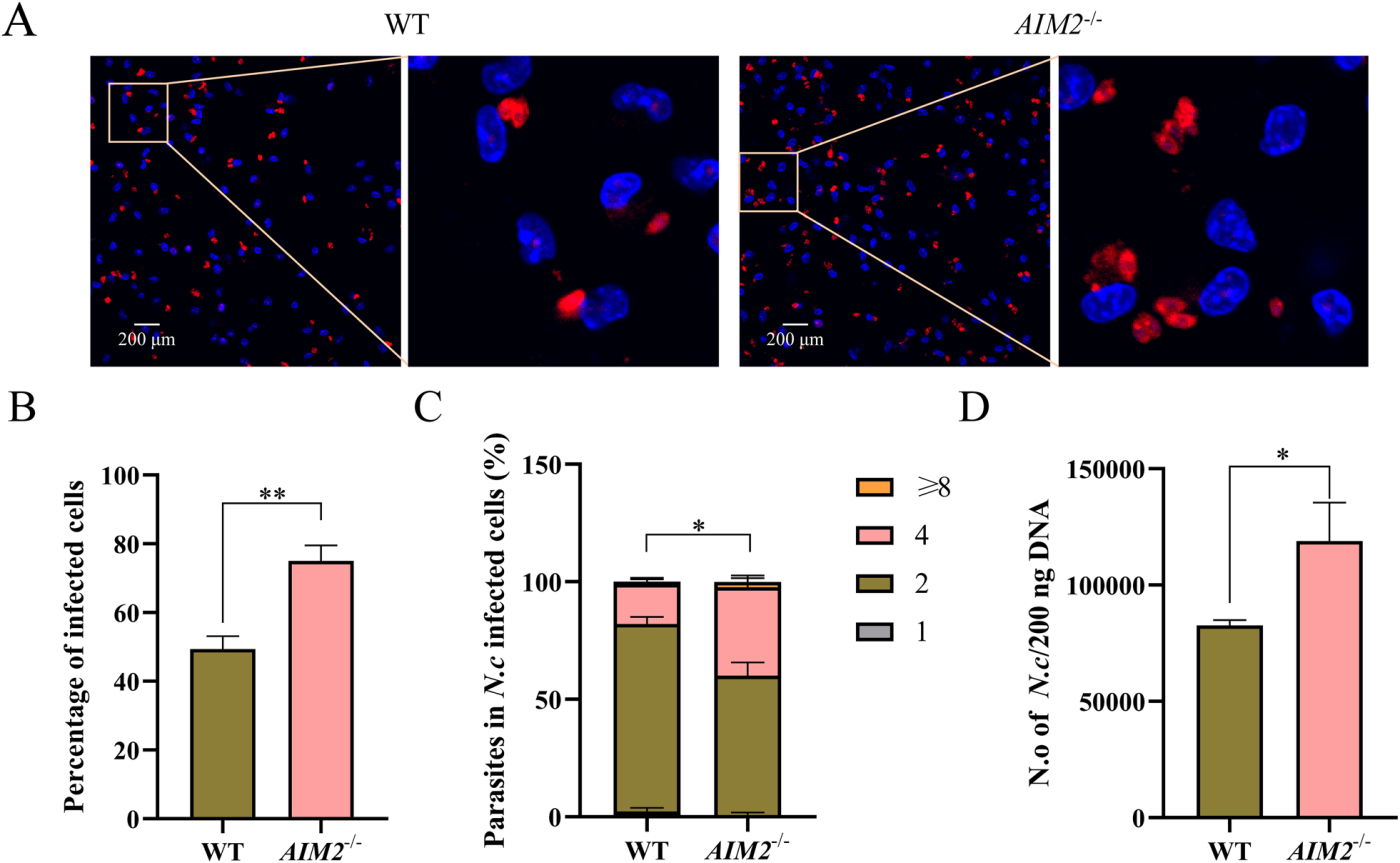
**AIM2 inflammasome resists *N. caninum* proliferation in mice macrophages.** WT and *AIM2*^*−*/*−*^ PMϕs were infected with *N. caninum* for 24 h. **A** The images of IFA. Nuclei were shown in blue (DAPI), *N. caninum* in red (SAG1), scale bars = 200 μm. **B** The percentage of *N. caninum*-infected cells was assessed in each image. **C** Intracellular replication was measured by counting the number of tachyzoites in 100 parasitophorous vacuoles. **D** The number of *N. caninum* tachyzoites was detected by qPCR. Data are displayed as mean ± SD of three independent experiments (*n *= 3). *P < 0.05, **P < 0.01 (t test).

To explore the role of AIM2 inflammasome in controlling *N. caninum* infection, we assessed the percentage of *N. caninum*-infected cells and the proliferation of *N. caninum* in WT and *AIM2*^*−*/*−*^ PMϕs. IFA and Giemsa staining revealed that the infection rate and the number of *N. caninum* tachyzoites were significantly increased in *AIM2*^*−*/*−*^ PMϕs compared with WT PMϕs (*P* < 0.05) (Figure 5A–C and Additional file 4A–C). qPCR analysis further showed that the number of *N. caninum* tachyzoites in *AIM2*^*−*/*−*^ PMϕs was increased by 43.85% compared with that in WT PMϕs (*P* < 0.05) (Figure 5D). These results indicated that the activation of AIM2 inflammasome inhibited *N. caninum* proliferation in mouse PMϕs.

## *N. caninum* infection upregulates AlM2 expression in mice

**Figure 6:**
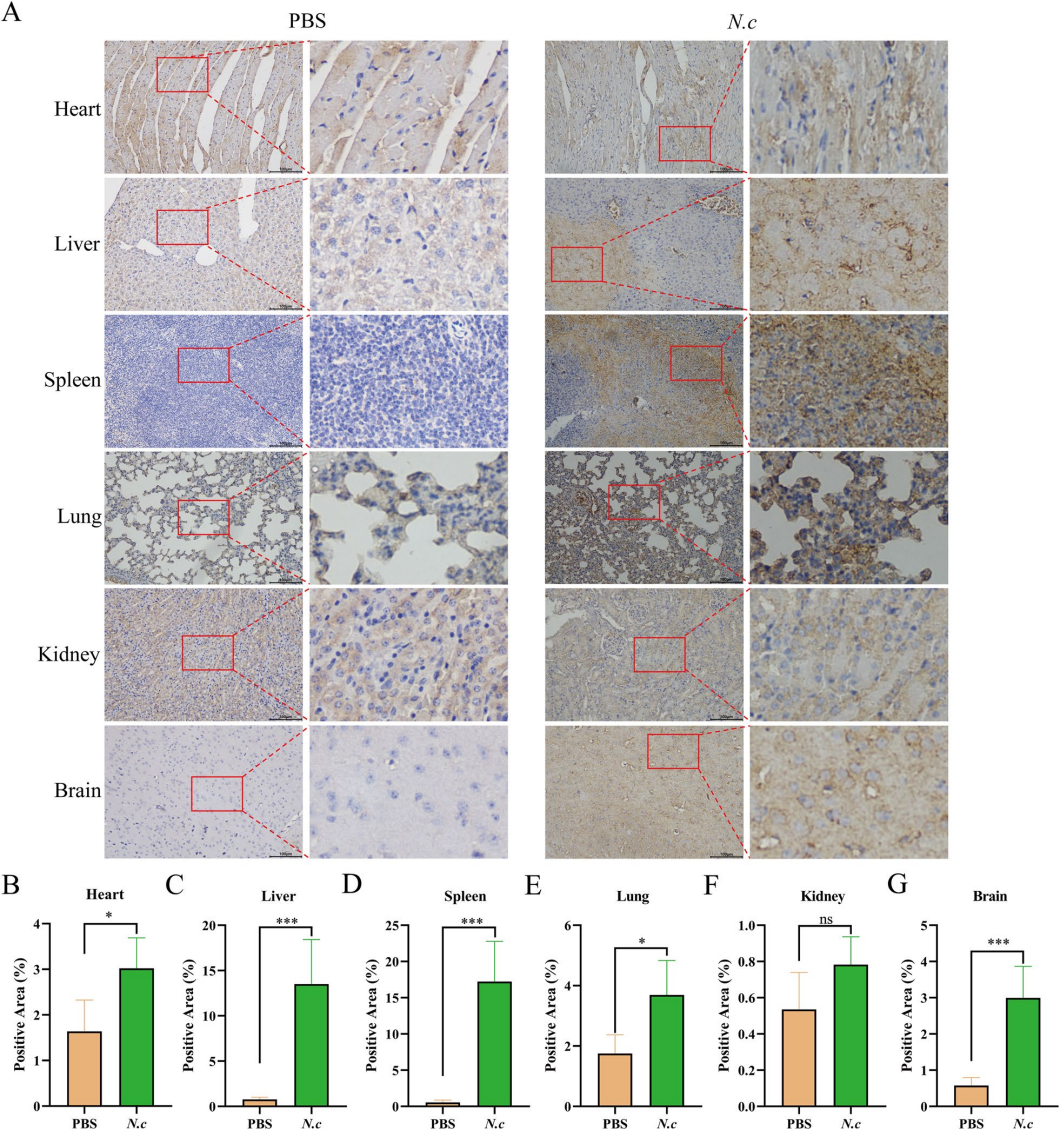
***Neospora caninum*** **infection upregulates AIM2 expression in mice tissues**. WT mice (*n *= 5) were intraperitoneally injected with 2 × 10^7^ *N. caninum* tachyzoites or an equal volume of PBS, and tissues were collected on day 5 post-infection. **A** AIM2 expression in tissues was detected by IHC, magnification 200 ×, scale bars = 100 μm. **B–G** The quantification of AIM2-positive staining in the heart **B** liver **C** spleen **D** lung **E** kidney **F** and brain **G** was evaluated using ImageJ. Data are displayed as mean ± SD of three independent experiments (*n *= 3). ns, no significant difference, *P < 0.05, ***P < 0.001 (t test).

To confirm AIM2 inflammasome activation in vivo, we examined AIM2 expression in various tissues from *N. caninum*-infected mice. IHC analysis revealed that AIM2 expression levels were significantly upregulated in the heart (*P* < 0.05), liver (*P* < 0.001), spleen (*P* < 0.001), lung (*P* < 0.05), and brain (*P* < 0.001) of *N. caninum*-infected group compared with PBS group, whereas no significant difference in AIM2 expression was observed in the kidney (Figure 6). These results indicated that *N. caninum* infection induced the upregulation of AIM2 expression in mice.

## AIM2 inflammasome triggers *N. caninum*-induced inflammatory responses in mice

**Figure 7:**
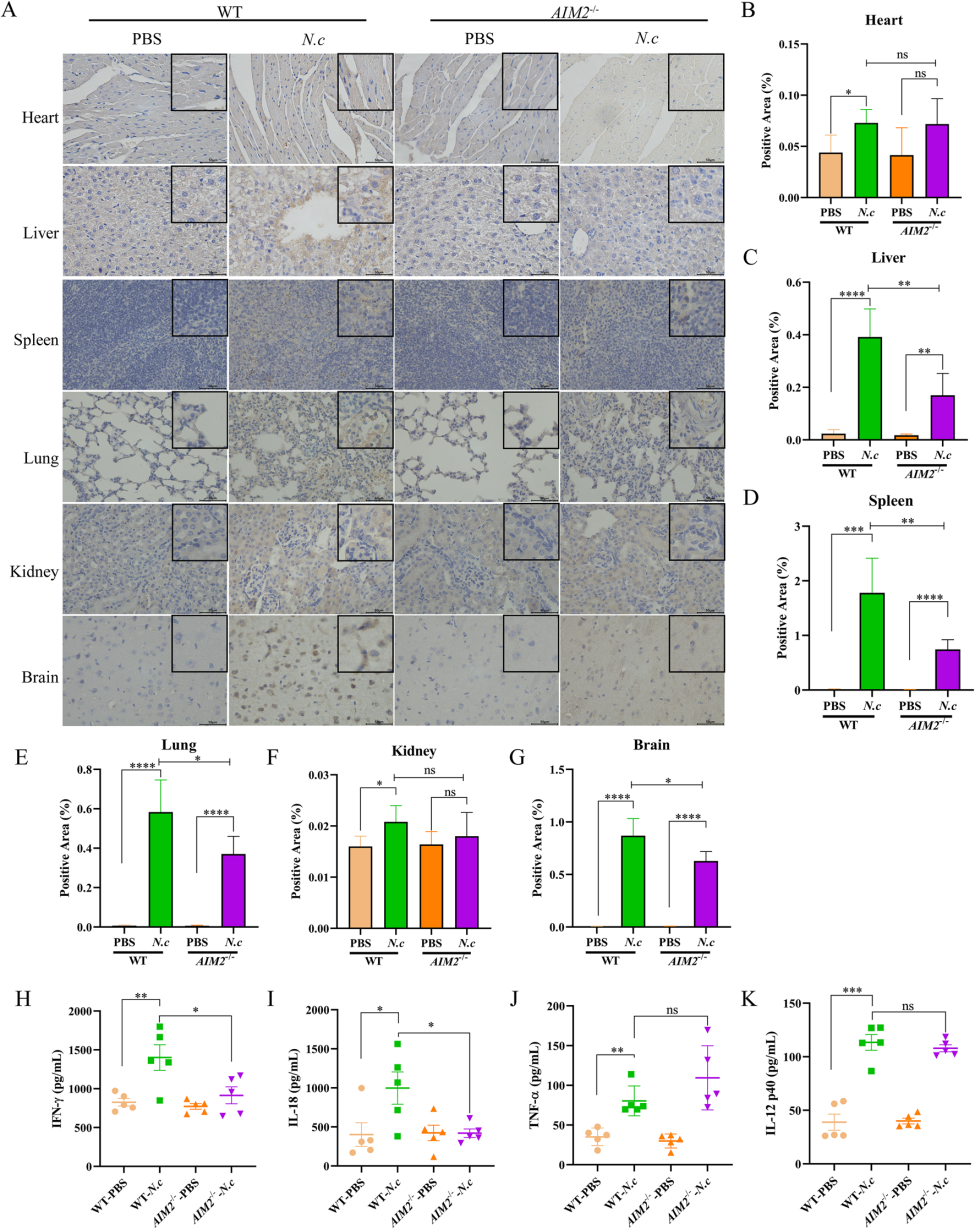
***AIM2*** **deficiency reduces macrophage recruitment and pro-inflammatory cytokine production in** ***N. caninum*-infected mice.** WT and *AIM2*^*−*/*−*^ mice (*n *= 5) were intraperitoneally injected with 2 × 10^7^ *N. caninum* tachyzoites or an equal volume of PBS, and tissues and serum were collected on day 5 post-infection. **A** Macrophages in mice tissues were detected by IHC using an anti-F4/80 antibody. Magnification 400 ×, scale bars = 50 μm. **B–G** The F4/80-positive area in the heart, liver, spleen, lung, kidney, and brain was quantified using ImageJ. **H–K** The levels of IFN-γ, IL-18, TNF-α, and IL-12 p40 in serum were measured by ELISA. Data are displayed as mean ± SD of three independent experiments (*n *= 3). ns, no significant difference, *P < 0.05, **P < 0.01, ***P < 0.001, and **** *P* < 0.0001 (t-test and one-way ANOVA).

To investigate the regulatory effect of AIM2 inflammasome in *N. caninum*-induced inflammatory responses in vivo, WT and *AIM2*^*−*/*−*^ mice were intraperitoneally injected with *N. caninum* tachyzoites, macrophage recruitment in tissues was assessed by F4/80 IHC, and serum cytokine levels were measured by ELISA. Compared with the PBS group, *N. caninum*-infected mice exhibited significantly increased macrophage recruitment in the heart, liver, spleen, lung, kidney, and brain (Figure 7A–G), and elevated serum levels of IFN-γ (Figure 7H), IL-18 (Figure 7I), TNF-α (Figure 7J), and IL-12 p40 (Figure 7K). Notably, *AIM2* deficiency significantly attenuated macrophage recruitment in the liver (*P* < 0.01), spleen (*P* < 0.01), lung (*P* < 0.05), and brain (*P* < 0.05) compared with WT mice, whereas no significant differences were observed in the heart and kidney (Figure 7A–G). Moreover, serum levels of IFN-γ (*P* < 0.05) (Figure 7H) and IL-18 (*P* < 0.05) (Figure 7I) were significantly reduced in *AIM2*^*−*/*−*^ mice compared with WT mice following *N. caninum* infection, while the levels of IL-12 and TNF-α had no differences (Figure 7J, K). These results demonstrated that the AIM2 inflammasome regulated inflammatory responses during *N. caninum* infection in vivo.

## AIM2 inflammasome exacerbates tissue damage and inhibits *N. caninum* proliferation in mice

**Figure 8:**
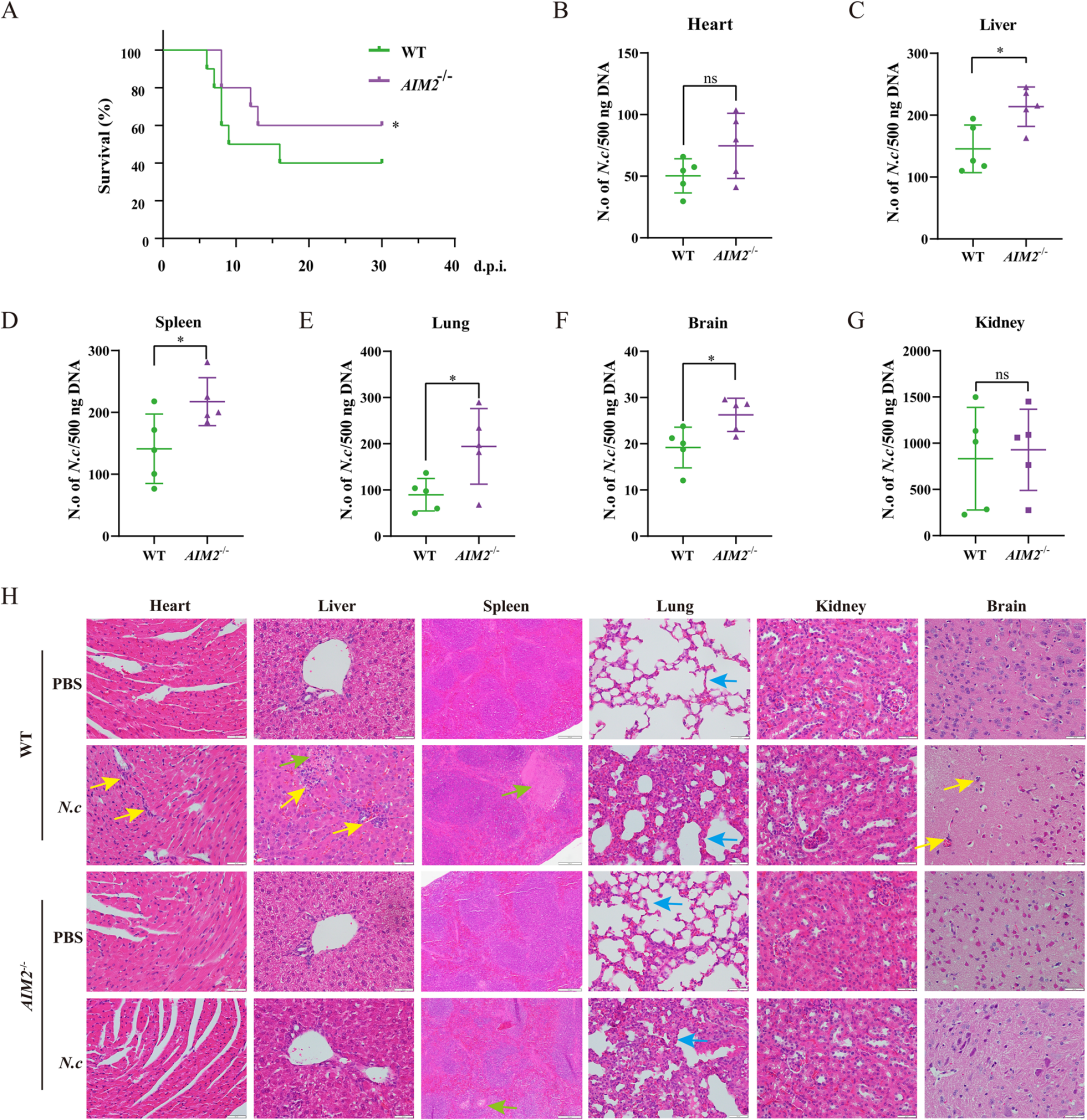
***AIM2*** **deficiency alleviates tissue injury and accelerates parasite proliferation in** ***N. caninum*-infected mice.** WT and *AIM2*^*−*/*−*^ mice were infected with 2 × 10^7^ *N. caninum* tachyzoites. **A** Survival curves of WT and *AIM2*^*−*/*−*^ mice (*n *= 10). Statistical analysis was performed using the log-rank test. **B–G** Parasite load in the heart, liver, spleen, lung, brain, and kidney was measured by qPCR (*n *= 5). **H** Histopathological changes in heart, liver, spleen, lung, kidney, and brain were assessed by **H, E** staining (*n *= 5). Magnification 400 ×, scale bars = 20 μm. Yellow arrows indicate inflammatory cells, blue arrows indicate alveolar walls, and green arrows indicate necrotic foci. Data are displayed as mean ± SD of three independent experiments (*n *= 3). ns, no significant difference, *P < 0.05 (t-test).

To further evaluate the role of the AIM2 inflammasome in *N. caninum* infection in vivo, we compared survival rates, tissue pathology, and parasite loads between WT and *AIM2*^*−*/*−*^ mice following intraperitoneal infection. *N. caninum*-infected *AIM2*^*−*/*−*^ mice exhibited a higher survival rate compared with WT mice (Figure 8A). In *AIM2*^*−*/*−*^ mice, parasite loads were markedly higher than in WT mice, with increases of 48.5% in the heart (*P* > 0.05), 46.7% in the liver (*P* < 0.05), 53.9% in the spleen (*P* < 0.05), 116.8% in the lung (*P* < 0.05), and 36.9% in the brain (*P* < 0.05) (Figure 8B–F), while there was no significant difference in the kidney between the two groups (Figure 8G). H&E staining showed that *N. caninum*-infected WT mice exhibited marked inflammatory cell infiltration in the heart, liver, and brain, and severe necrotic foci in the liver and spleen, and disruption of normal lymphoid architecture with effacement of the marginal zone between red and white pulp in the spleen, thickened alveolar walls and alveolar atrophy in the lung, widened renal interstitial spaces in the kidney (Figure 8H). While *N. caninum*-infected *AIM2*^*−*/*−*^ mice exhibited markedly reduced inflammatory cell infiltration in the heart, liver, and brain, and decreased necrotic area in the liver and spleen, but more severe loss of demarcation between red and white pulp in the spleen compared with WT mice (Figure 8H). These results indicated that the activation of AIM2 inflammasome exacerbated tissue damage and inhibited *N. caninum* proliferation in vivo.

## Discussion

Bovine abortion represents a major economic burden for the global livestock industry, and *N. caninum* is widely recognized as a leading etiological agent of reproductive failure in cattle [3]. Mounting evidence indicates that *N. caninum* infection triggers a complex host immune response, with innate immunity playing a critical role in the pathogenesis of neosporosis [39–42]. Therefore, identifying the innate immune pathways that balance host defense and immunopathology is crucial for the development of targeted control strategies against neosporosis.

The role of inflammasomes in regulating pathogen infection has gained increasing attention. Studies have shown that the NLRP3 inflammasome is activated by *N. caninum* in both murine and bovine cells, contributing to host anti-parasitic immunity through the maturation and secretion of IL-1β and IL-18 [32, 33]. Additionally, the NLRC4 inflammasome has also been implicated in the host response against *N. caninum* in mouse macrophages [43]. The AIM2 inflammasome is a pivotal cytosolic DNA-sensing component of the innate immune system. Its activation drives caspase-1 cleavage, pyroptosis, and the secretion of pro-inflammatory cytokines IL-1β and IL-18, which are core processes involved in initiating anti-pathogen or pathological inflammation in various infectious diseases, including those caused by *S. pneumoniae*, *L. monocytogenes*, *M. bovis*, hepatitis B virus, and *T. gondii* [8, 9, 11, 44, 45]. In this study, we demonstrated that *N. caninum* infection activated the AIM2 inflammasome in mouse peritoneal macrophages and a murine infection model, and AIM2 inflammasome activation restricted parasite proliferation but also exacerbated tissue pathology, revealing an immunological trade-off with significant implications for neosporosis.

A critical prerequisite for AIM2 inflammasome activation is the release of microbial double-stranded DNA into the host cytosol, a process increasingly attributed to GBPs. Accumulating evidence indicates that GBPs not only recognize pathogen-containing vacuoles but also execute direct membranolytic attacks that breach microbial integrity [18, 19, 21, 22]. For instance, GBP1 has been implicated in perforating *L. monocytogenes* phagosomal membranes, facilitating cytosolic access of listerial DNA [46]. Similarly, GBP1, GBP2, and GBP4 localize to *S. flexneri* membranes to restrict infection spread [18]. Direct evidence also supports the role of GBPs in promoting AIM2 inflammasome activation. GBP2 and GBP5 disrupt bacterial membranes and enhance AIM2 inflammasome assembly during *Francisella novicida* and *B. abortus* infections [24, 38]. Our previous work identified GBP2 and GBP5 as the most highly upregulated GBPs in *N. caninum*-infected mouse PMϕs [31]. We therefore hypothesized that GBP2 or GBP5 translocates to the parasite membrane and disrupts its structure, thereby providing the DNA ligand required for AIM2 sensing. Here, we showed that GBP5, but not GBP2, robustly colocalized with the *N. caninum* membrane protein NcSAG1 and induced parasite membrane lysis. Overexpression of GBP5 enhanced AIM2 inflammasome activation, whereas GBP5 knockdown attenuated it, suggesting that GBP5 is both necessary and sufficient for AIM2 inflammasome activation during *N. caninum* infection. This aligns with studies of *T. gondii*, where GBP1, 2, 3, 5, 6, 7, and 9 disrupt the PVM and parasite plasma membrane to liberate DNA [16, 21, 22].

We further demonstrated that the AIM2 inflammasome played a dual role in host defense and immunopathology during *N. caninum* infection in mice. *AIM2* deficiency led to increased parasite proliferation in macrophages and higher parasite burdens in multiple organs, indicating that the AIM2 inflammasome contributes to host resistance against *N. caninum*. This finding is consistent with the protective role of AIM2 inflammasome against various pathogens, including *S. pneumoniae*, *L. monocytogenes*, *M. tuberculosis*, *M. bovis*, *Brucella*, murine cytomegalovirus, and *T. gondii* [7–10, 45, 47, 48]. Pro-inflammatory cytokines are widely recognized as essential for combating pathogen infections [49, 50]. IL-12 and IFN-γ drive Th1-mediated anti-*N. caninum* immunity in cattle and mice [51, 52], and IL-18 potentiates IFN-γ production and restricts *N. caninum* replication [33]. Therefore, in our study, the reduced secretion of IL-1β, IL-18, and IFN-γ in *AIM2*^*−*/*−*^ mice likely compromised immune activation and parasite control, as these cytokines are critical for driving Th1 responses and enhancing macrophage effector functions against *N. caninum*.

Concurrently, our study demonstrated that *N. caninum*-infected *AIM2*^*−*/*−*^ mice exhibited reduced IL-1β, IFN-γ, and IL-18 release, and inflammatory cell recruitment in the liver, spleen, lung, and brain, diminished necrotic tissue damage in the liver and spleen, and a significantly higher survival rate compared with WT mice. This finding demonstrated that AIM2 inflammasome activation exacerbated *N. caninum*-induced immunopathology in mice, in line with studies linking AIM2 activation to tissue injury via macrophage recruitment and pro-inflammatory cytokine secretion [12, 53, 54]. For instance, IL-18 and IFN-γ are known to induce potent inflammatory responses, and their overproduction has been linked to lethal immunopathology in acute toxoplasmosis [55, 56]. *Cronobacter sakazakii* activates the AIM2 inflammasome in mammary epithelial cells, leading to TNF-α, IL-1β, and IL-6 secretion and subsequent tissue injury [57]. During *Plasmodium* or *L. (V.) braziliensis* infection, AIM2 activation triggers macrophage recruitment and systemic IL-1β-driven inflammation [12, 58]. Therefore, AIM2 may mediate the excessive pro-inflammatory cytokine production and macrophage infiltration observed in *N. caninum*-infected WT mice, which may be the primary driver of severe liver and spleen tissue injury. Our observation that AIM2 deficiency leads to increased parasite burden yet improved survival outcomes further underscores this immunological trade-off. Similar to our study, *NOD2* deficiency in mice also results in reduced inflammation and improved survival, accompanied by increased parasite burden [59]. These emphasize the importance of balancing the host's defense mechanisms with immune pathological responses, which must be carefully taken into account when designing immunomodulatory therapies for neosporosis. The goal in such treatments is to suppress pathological inflammation without eliminating the protective anti-parasite immunity.

Notably, NLRP3 inflammasome activation has previously been shown to restrain *N. caninum* proliferation in murine and bovine macrophages [32, 33], and our work further identified AIM2 as an additional key inflammasome module in anti-*N. caninum* innate immunity. Importantly, *AIM2* deficiency did not significantly alter NLRP3 protein expression or aggregation in *N. caninum*-infected macrophages, suggesting that the impaired host defense observed in *AIM2*^*−*/*−*^ macrophages is specifically attributable to the loss of AIM2 inflammasome signaling. This finding underscores the crucial role of AIM2 as a cytosolic sentinel for *N. caninum* in mice. However, given that cattle are the primary host for neosporosis and that immune responses differ between species, caution is warranted when extrapolating these findings to bovines. Importantly, several studies have reported that the bovine genome also encodes GBP1, GBP2, GBP4, GBP5, and GBP6, and bovine GBPs share high structural and sequence homology with human and murine GBPs [26, 28–30]. Bovine GBP5 confers resistance to herpesvirus 1 infection [26]. Moreover, studies have also reported that *N. caninum* infection elicits similar immune responses in murine and bovine cells, including the activation of NLRP3 inflammasome, NOD2, TLR3, and NF-κB signaling pathways [32, 33, 39, 41, 59–61]. Therefore, these observations raise the possibility that a GBP5-AIM2 inflammasome axis, analogous to that characterized in mice, may also drive inflammatory responses during *N. caninum* infection in cattle. Future studies should validate this axis in bovine macrophages.

**Figure 9:**
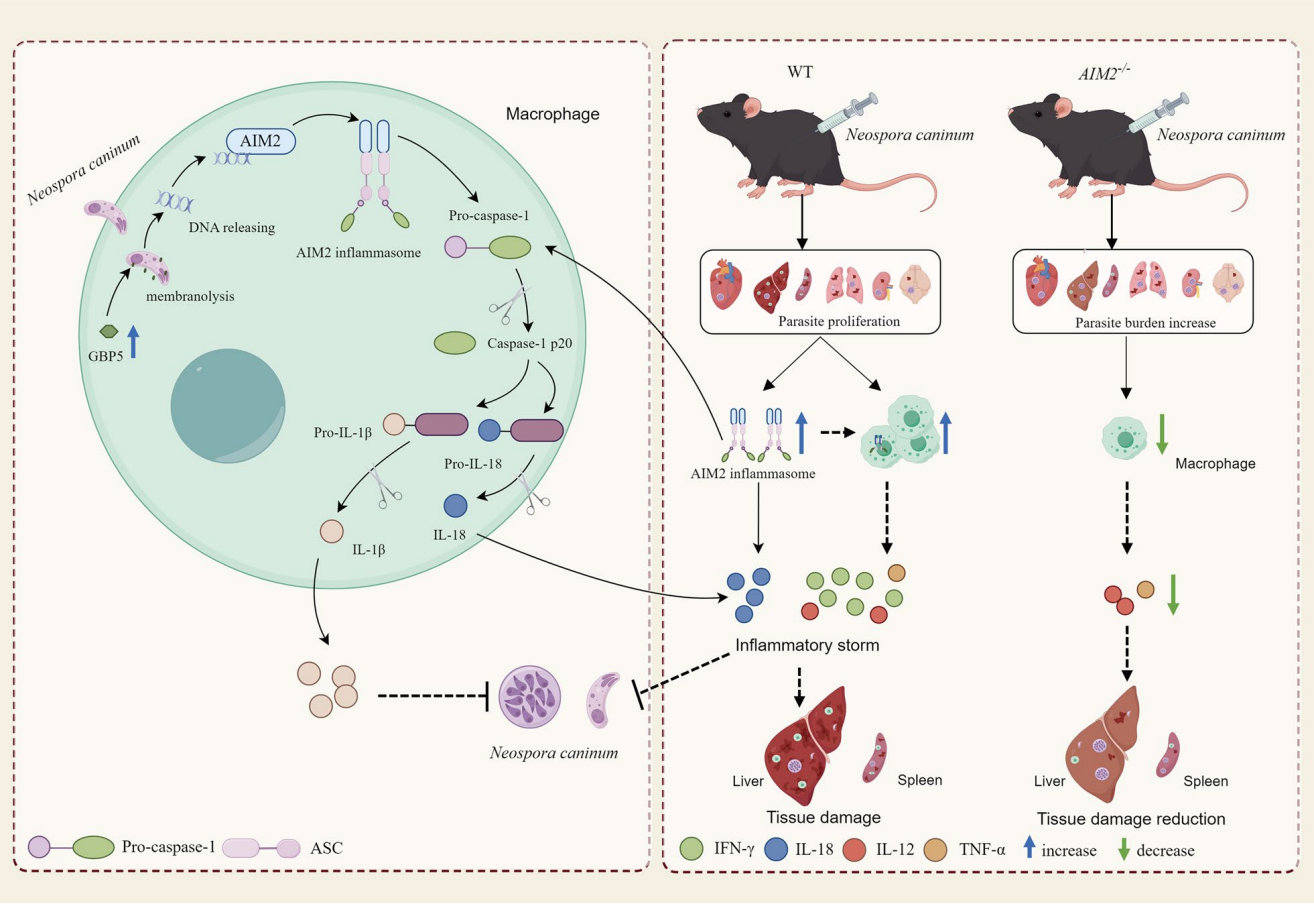
**A model of the activation and role of the AIM2 inflammasome during *N. caninum*** **infection in vitro and in vivo.** GBP5 locates on the parasite plasma membrane to trigger AIM2 inflammasome activation during *N. caninum* infection, AIM2 inflammasome promotes macrophage recruitment and induces the secretion of IL-1β, IFN-γ, and IL-18, contributing to the control of parasite proliferation while also causing severe tissue damage in the liver and spleen.

In conclusion, our findings demonstrate that GBP5 promotes AIM2 inflammasome activation during *N. caninum* infection. The AIM2 inflammasome promotes macrophage recruitment and induces the secretion of IL-1β, IFN-γ, and IL-18, thereby contributing to the control of parasite proliferation but also driving disease severity (Figure 9). These findings advance our understanding of innate immunity to apicomplexan parasites and suggest that targeting the GBP5-AIM2 axis may offer a strategy to modulate inflammation in neosporosis. However, given the species-specific differences in immune responses, further work in bovine systems is essential to assess the translational potential of these observations for improving animal health and reducing the economic impact of neosporosis in cattle.

## Supplementary Information

**Additional file 1 Sequences of forward and reverse primers used for PCR amplification.**

**Additional file 2 AIM2 deficiency does not alter NLRP3 inflammasome activation in *N. caninum*-infected macrophages.** WT and AIM2^-/-^ PMϕs were infected with N. caninum for 24 h. (A) NLRP3 aggregation was detected by IFA. Nuclei were stained with DAPI (blue), NLRP3 (red), scale bars = 20 μm. Zoomed images correspond to the areas within the orange boxes. (B) NLRP3 protein expression was detected by western blot. (C) NLRP3 protein level was quantified using ImageJ. Data are displayed as mean ± SD of three independent experiments (*n* = 3). ns, no significant difference (t- test).

**Additional file 3 Quantitative analysis of proteins.** (A-H) Protein levels were quantified using ImageJ. Data are displayed as mean ± SD of three independent experiments (*n *= 3). ns, no significant difference, *P < 0.05, **P < 0.01, ***P < 0.001 (t-test and one-way ANOVA).

**Additional file 4 AIM2 inflammasome resists *N. caninum* infection in mice macrophages**. (A) The figures of Giemsa staining. Red arrows indicate the parasitophorous vacuole. (B) The percentage of N. caninum-infected cells was assessed in each image. (C) Intracellular replication was measured by counting the number of tachyzoites in 100 parasitophorous vacuoles.

## Data Availability

All relevant data are included in the manuscript and Additional files.
