# Supplementary material for: GBP5-triggered AIM2 inflammasome drives host defense and exacerbates disease severity during Neospora caninum infection
Source: Vet Res. 2026 Jun 20;57:113. doi: 10.1186/s13567-026-01769-z (PMC13283329; doi:10.1186/s13567-026-01769-z)
Supplement: Supplementary file 1 — Additional file 1 Sequences of forward and reverse primers used for PCR amplification. [file 13567_2026_1769_MOESM1_ESM.docx]

Addition file 1. Sequences of forward and reverse primers used for PCR amplification

| Gene | Forward Primer | Reverse Primer |
| --- | --- | --- |
| GAPDH | AGGGAGCTAAAACCATCCAG | ATCTTCAAGGCTTTTCCCCAC |
| AIM2 | CCACATCACGGAGGAAGAACT | GAGGCAGCAGAGCAGTTTTC |
| Hprt | CAGTCCCAGCGTCGTGATTA | TGGCCTCCCATCTCCTTCAT |
| Rpl13a | cctgctgctctcaaggttgtt | CGATAGTGCATCTTGGCCTTT |
| Tbp | CTACCGTGAATCTTGGCTGTAA | GTTGTCCGTGGCTCTCTTATT |
| Ppia | ATGGCAAGCATGTGGTCTTTGG | ATCTTCTTGCTGGTCTTGCCAT |
