# Supplementary figures and images for: GBP5-triggered AIM2 inflammasome drives host defense and exacerbates disease severity during Neospora caninum infection
Source: Vet Res. 2026 Jun 20;57:113. doi: 10.1186/s13567-026-01769-z (PMC13283329; doi:10.1186/s13567-026-01769-z)

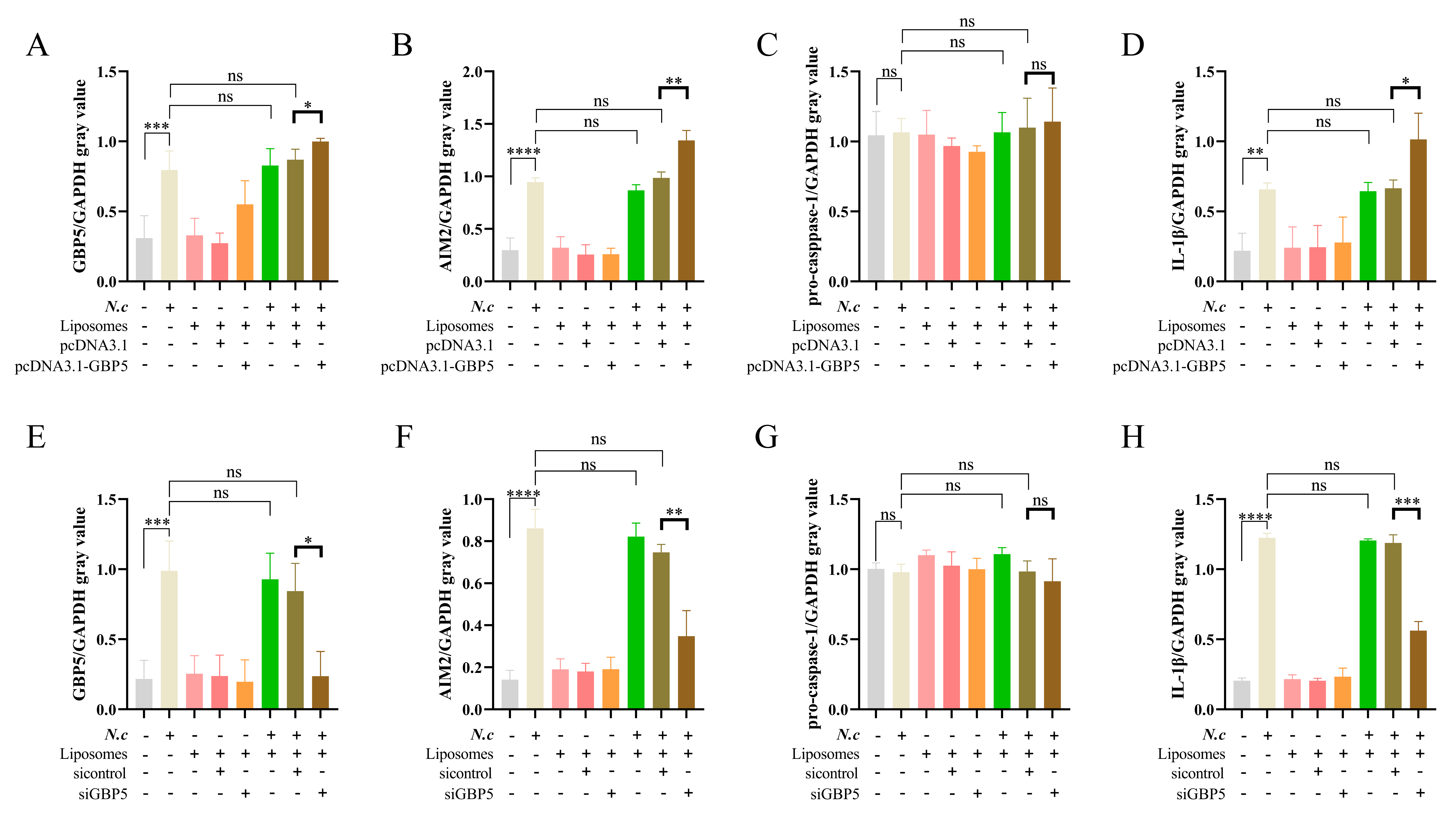

Supplement: Supplementary file 3 — Additional file 3 Quantitative analysis of proteins. (A-H) Protein levels were quantified using ImageJ. Data are displayed as mean ± SD of three independent experiments (n = 3). ns, no significant difference, *P < 0.05, **P < 0.01, ***P < 0.001 (t-test and one-way ANOVA). [file 13567_2026_1769_MOESM3_ESM.tif]

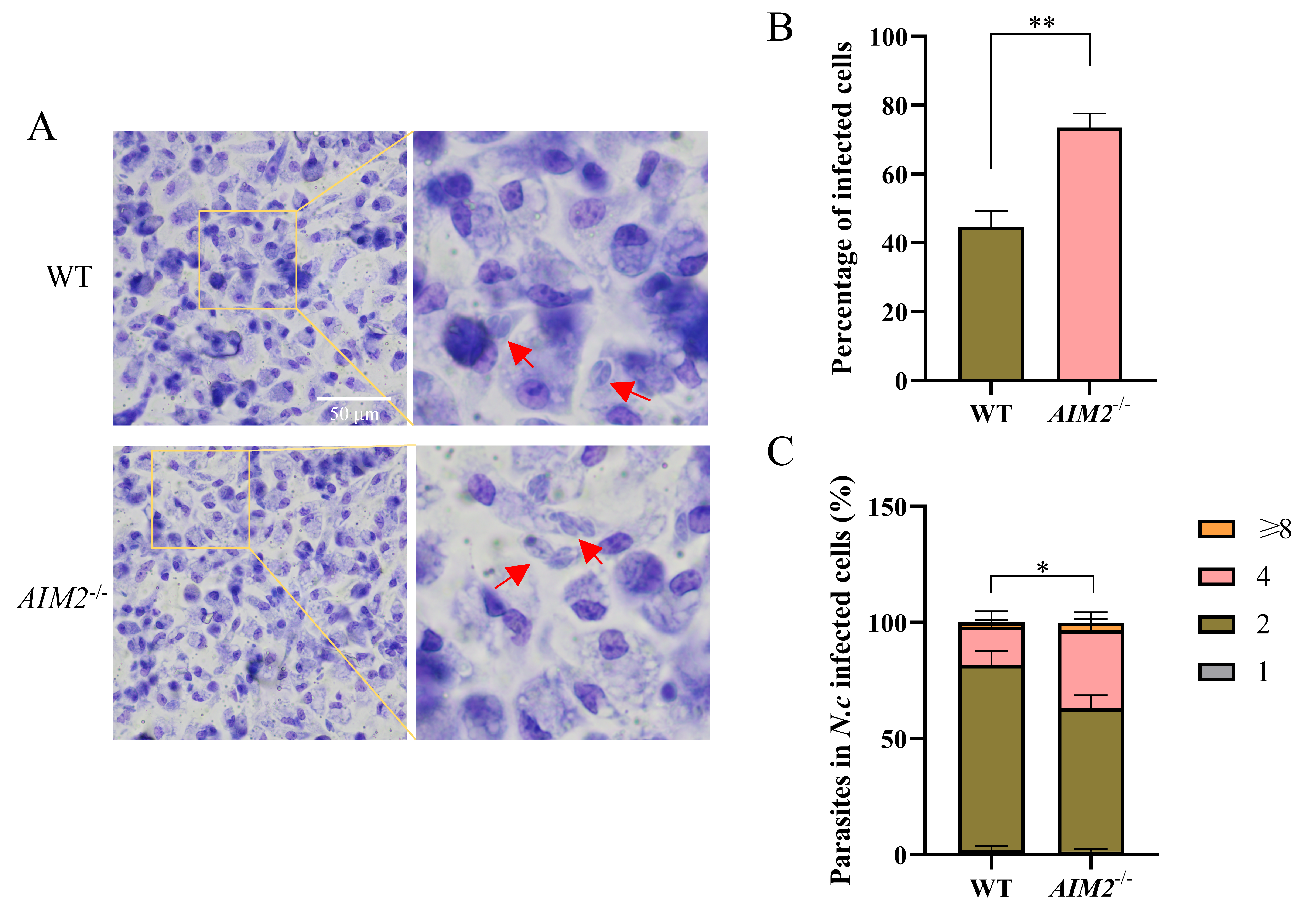

Supplement: Supplementary file 4 — Additional file 4 AIM2 inflammasome resists N. caninum infection in mice macrophages. (A) The figures of Giemsa staining. Red arrows indicate the parasitophorous vacuole. (B) The percentage of N. caninum-infected cells was assessed in each image. (C) Intracellular replication was measured by counting the number of tachyzoites in 100 parasitophorous vacuoles. [file 13567_2026_1769_MOESM4_ESM.tif]
